# Study of Blood Biomarkers in Athletes with Lower Gastrointestinal Symptoms After an Ultra-Trail Race

**DOI:** 10.3390/jcm14031024

**Published:** 2025-02-06

**Authors:** Joshua Teyssier, Sébastien Perbet, Bruno Pereira, Stéphane Bergzoll, Mathieu Kuentz, Julie Durif, Vincent Sapin, Matthieu Jabaudon, Damien Bouvier

**Affiliations:** 1 Biochemistry and Molecular Genetic Department, Clermont-Ferrand Teaching Hospital, 63000 Clermont-Ferrand, France; joshua.teyssier@yahoo.fr (J.T.); j_durif@chu-clermontferrand.fr (J.D.); vsapin@chu-clermontferrand.fr (V.S.); 2 Anesthesiology and Critical Care Department, Clermont-Ferrand Teaching Hospital, 63000 Clermont-Ferrand, France; perbetseb@hotmail.com (S.P.); mjabaudon@chu-clermontferrand.fr (M.J.); 3 Biostatistics Unit (DRCI), Clermont-Ferrand Teaching Hospital, 63000 Clermont-Ferrand, France; bpereira@chu-clermontferrand.fr; 4 Emergency Department, Renaison Clinic, 42300 Roanne, France; stephane.bergzoll@gmail.com; 5 Biology Department, Aurillac Hospital, 15000 Aurillac, France; matkuentz@gmail.com

**Keywords:** digestive symptoms, ultra-trail, I-FABP, IL-10, IL-1Ra, IL-6

## Abstract

**Background/Objectives**: To investigate the value of intestinal fatty acid-binding protein (I-FABP), D-Lactate, interleukin-6 (IL-6), interleukin-10 (IL-10), interleukin-1 receptor antagonist (IL-1Ra), tumor necrosis factor-alpha (TNF-alpha), lactate dehydrogenase (LDH), alanine aminotransferase (ALT), aspartate aminotransferase (AST), creatine kinase (CK), electrolytes and creatinine in athletes with lower gastrointestinal symptoms in a cohort of ultra-trailers. **Methods**: This is a prospective study set in the ultra-trail of Puy Mary Aurillac, a 105 km race. Athletes included were given two questionnaires to collect demographic data and clinical signs related to the race. Blood samples were also collected before and 1 h after the race. Biomarker results were interpreted according to the occurrence of exercise-induced lower gastrointestinal symptoms, and whether the race was completed or forfeited. **Results**: Of the 76 runners included, 35 (45.5%) presented lower gastrointestinal symptoms. Runners that presented these symptoms had significantly higher IL-10 concentrations (8.7 pg/mL (interquartile range (IQR): 4.2–1.6)) when compared to runners without symptoms (4.8 pg/mL (IQR: 2.4–9)) (*p* = 0.01). The pre/post-race amplitude of IL-1Ra variation was greater in the group of runners with lower gastrointestinal symptoms (median: +231% (IQR: 169–551)) compared to runners without symptoms (median: +172% (IQR: 91–393)) (*p* = 0.04). Finally, the 13 (16.9%) runners who forfeited the race displayed lower AST (*p* < 0.001), LDH (*p* = 0.002) and IL-6 (*p* = 0.002) concentrations, compared to runners who finished the race. These lower concentrations were independent from running time. **Conclusions**: IL-10 and IL-1Ra could be associated with the occurrence of lower gastrointestinal symptoms.

## 1. Introduction

Ultra-trail, initially defined as races of over 80 km, was born in the 1970s and has continued to develop ever since. This discipline consists of races exceeding 80 km (ultra-distance), run in natural environments on courses marked out by the organizers. Often, when these races are run in mountainous terrain, the vertical drop continent plays an important role in the difficulty of the event. These events can be run all year round, with winter events also available. One of the most popular distances is the 100 miles, with world-famous trails around the globe. Since 2010, a world cup circuit has been created with “world tour” events on every continent, featuring different disciplines depending on the distance. Being an endurance discipline, trail running performance is mainly determined by aerobic capacity, as well as by muscular strength and body composition for distances around 100 km [1].

Exercise-induced gastrointestinal symptoms are common in endurance athletes. Around 40% of runners describe upper gastrointestinal symptoms (gastroesophageal reflux disease, nausea, vomiting, etc.) and 70% of runners describe lower gastrointestinal symptoms during long races such as ultra-trails [2,3]. The mechanisms underlying the observed symptoms are certainly multi-factorial and may be explained by cellular suffering due to mesenteric ischemia and inflammation [4]. Physiologically, during physical exercise, the increased activity of the sympathetic nervous system redistributes blood flow from the splanchnic organs to the working muscles [5]. In cases of prolonged duration and/or intensity, splanchnic blood flow can be reduced by 80% or more [6]. A significant reduction in splanchnic blood flow frequently leads to functional or, in rare cases, pathological gastrointestinal ischemia. This ischemia associated with reduced vagal activity probably leads to changes in motility and absorption [7]. Furthermore, a major increase in inflammatory cytokines is observed during physical exercise, particularly during intense, long-duration exercise [8,9].

Several blood biomarkers can be proposed to reflect the pathophysiological changes observed in the context of mesenteric ischemia. Among these biomarkers, I-FABP (fatty acid-binding protein), a 15 kDa cytosolic protein present in mature enterocytes in the villi of the small intestine and involved in the absorption and metabolism of fatty acids, constitutes an early marker of mesenteric ischemia [10,11]. I-FABP is released into the bloodstream in the event of enterocyte damage affecting cell membrane integrity [12,13]. I-FABP levels increase after marathon running and rise further in case of exercise-associated collapse and hyperthermia [14]. D-Lactate, a molecule of bacterial origin, has also been described as a biomarker of intestinal suffering [15]. Regarding the inflammatory component, significant increases in interleukin-6 (IL-6), interleukin-10 (IL-10), interleukin-1 receptor antagonist (IL-1Ra) and tumor necrosis factor-alpha (TNF-alpha) have been reported in such sporting competition [9]. Other biomarkers of cellular suffering have been shown to significantly increase in this context of highly intensive muscular demand; these markers include lactate dehydrogenase (LDH), alanine aminotransferase (ALT), aspartate aminotransferase (AST) and creatine kinase (CK) [16,17]. Finally, the measurements of electrolytes and creatinine highlight dehydration [18].

In this context, the aim of this study was to investigate the value of biomarkers I-FABP, D-Lactate, IL-6, IL-10, IL-1Ra, TNF-alpha, LDH activity, ALT activity, AST activity, CK activity, electrolytes and creatinine in athletes presenting lower gastrointestinal symptoms after an ultra-trail. We hypothesize that certain markers show significantly higher blood concentrations in athletes with digestive signs. A secondary endpoint was to evaluate the evolution of these biomarkers in athletes who did not complete the race.

## 2. Materials and Methods

### 2.1. Study Design and Patients

Participants of the “ultra-trail du Puy Mary Aurillac” (UTPMA), a 105 km race with 5710 m of positive ascent, held on 19 June 2015, were recruited in this prospective, pathophysiological exploratory study that aimed to assess the evolution of biological markers associated with mesenteric ischemia (oxygen deprivation in the digestive organs) during ultra-trail running.

Athletes were first contacted by e-mail. The e-mail defined the study objectives and requirements, which included the collection of anthropometric data, dietary/fluid intake during the event, reporting of digestive symptoms, as well as blood sample collection and questionnaire completion. The inclusion criteria included being over 18 years of age and presenting a valid medical certificate attesting to the absence of contraindications for participation in the event.

The study adhered to the principles of the Declaration of Helsinki and received approval by the Ethics Committee Sud-Est VI (number AU1185) on 11 May 2015. It was conducted from 9 June 2015 to 20 June 2015.

Prior to the study, all participants underwent an individual interview in which they signed a written consent form. On the day preceding the race, participants completed an initial paper-based questionnaire designed to collect demographic and baseline data. A second questionnaire was completed after the race, in order to collect clinical signs and race-related data. Blood samples (15 mL per participant) were collected from all participants using sodium fluoride/potassium oxalate (NaF/KOx) tubes, serum sampling tubes (SST), and lithium heparin collection tubes both the day before the race and one hour post-race. The samples were stored in a shaded area near the participants’ gymnasium until analysis.

### 2.2. Procedures

The primary endpoint was to investigate the value of biomarkers I-FABP, D-Lactate, IL-6, IL-10, IL-1Ra, TNF-alpha, LDH activity, ALT activity, AST activity, CK activity, electrolytes and creatinine in athletes presenting lower gastrointestinal symptoms after an ultra-trail. A secondary endpoint was to evaluate the evolution of these biomarkers in athletes who did not complete the race.

Data collected were age, sex, weight, height, body mass index, run time, liquid ingestion in mL during race, cumulative distance over one week to estimate training level, withdrawal before the end of the race (yes or no) and number of km covered in case of withdrawal, presence of lower gastrointestinal symptoms defined by the presence of one of the following signs: abdominal pain, flatulence, urge to defecate, diarrhea and rectal bleeding [2].

### 2.3. Blood Assays

Venous blood samples were centrifuged at 2100× *g* for 15 min. Plasma and serum samples were stored at −80 °C until analysis.

Electrolytes (sodium, potassium, chloride), creatinine, ALT activity, AST activity, LDH activity and total CK activity concentration in heparinized plasma assays were performed using an Atellica^®®^ analyzer (Siemens, Munich, Germany) following the manufacturer’s recommendations. Indirect potentiometry was used for electrolytes and colorimetric methods for other analytes.

Serum IL-1Ra, IL-6, IL-10 and TNF-alpha concentrations were determined on Simple Plex Ella^®®^ Assay (ProteinSimple, San Jose, CA, USA) on Ella^®®^ instrument according to the manufacturers’ instructions. The Ella/Simple Plex technology is an automated immunoassay platform which utilizes a microfluidic design in a cartridge format.

Serum IFABP concentrations were determined on R-Plex Assay, a chemiluminescent assay (ref K151M4R-2, Meso Scale Discovery Inc., Rockville, MD, USA), on Meso QuickPlex SQ120^®®^ instrument according to the manufacturers’ instructions.

The concentrations of D-Lactate in NaF/KOx plasma were measured by the Enzyme-linked immunosorbent assay method using Abcam^®®^ kit (Abcam, Cambridge, UK) following the manufacturer’s instructions (ref AB83429-1001).

### 2.4. Statistics

Continuous data are expressed according to their statistical distribution as median and interquartile range. The assumption of normality was analyzed using the Shapiro–Wilk test.

To assess changes between pre-race and post-race, statistical paired tests were performed using the paired Student *t*-test or Wilcoxon test if the assumptions of the t-test were not met. Pitman’s test was used to analyze the equality of variances.

The Student t-test or the Mann–Whitney test were applied for comparisons between groups (i.e., digestive symptoms, withdrawal) of pre-race values, post-race values, and changes at post-race ((post-race − pre-race)/pre-race ∗ 100) in order to (1) investigate the value of these biomarkers in athletes presenting lower gastrointestinal symptoms after an ultra-trail (primary objective) and to (2) evaluate the evolution of these biomarkers in athletes who did not complete the race (secondary objective). The homoscedasticity was studied using the equality of variance Fisher–Snedecor test. Results are expressed using effect sizes and 95% confidence intervals and interpreted according to Cohen’s recommendations which define effect-size thresholds as small (ES: 0.2), medium (ES: 0.5) and large (ES: 0.8, “grossly perceptible and therefore large”). Multivariate analyses were conducted with multiple logistic regression to take into account possible confounders (i.e., run time, sex and age) for associations according to lower gastrointestinal symptoms (yes/no) and between withdrawal.

Statistical analyses were performed using the Stata software package (version 15, StataCorp, College Station, TX, USA). All statistical tests were carried out for a two-sided type I error at 5%. No correction for multiple testing was applied in the analysis of secondary outcomes. No imputation data approach was applied. The analyses concerning changes at post-race were performed only for patients with values at post-race. In addition to the comparisons between participants with and without missing data, Little’s MCAR (Missing Completely at Random) test was performed to assess whether the missing blood sample data followed a completely random pattern.

## 3. Results

### 3.1. Study Population

We recruited 120 runners at the start of the race. Post-race blood samples were available for only 76 runners (44 excluded: 22 refusal of blood test, 9 sampling failure, 13 lost to follow up). The description of these two groups is presented in Table 1.

### 3.2. Impact of the Race on Blood Biomarker Levels

Medians of AST activity, ALT activity, CK activity, creatinine, LDH activity, sodium, chloride, I-FABP, IL-10, IL-1Ra, IL-6, TNF-alpha and D-Lactate significantly increased after the race (Table 2). Median potassium significantly decreased after the race (Table 2).

### 3.3. Comparison of Biological Parameters with the Presence of Lower Gastrointestinal Symptoms

Pre-race medians of the measured biomarkers were not significantly different according to the presence or absence of lower gastrointestinal symptoms at the end of the race (Table 3).

A significant increase in median IL-10 after the race was observed in the group of runners with lower gastrointestinal symptoms (8.7 pg/mL (IQR: 4.2–1.6)) when compared to the group without lower gastrointestinal symptoms (4.8 pg/mL (IQR: 2.4–9)) (ES: 0.72 (95%CI: 0.25–1.19) (Table 3).

A significant difference was observed when comparing pre- and post-race variation of LDH activity between runners with lower gastrointestinal symptoms and runners without symptoms (medians at +105% (IQR: 57–157) and +68% (IQR: 46–102), respectively) (ES: 0.47 (95%CI: 0.00–0.93)) (Table 3). A significant difference was also observed for IL-1Ra between runners with lower gastrointestinal symptoms (median at +231% (IQR: 169–551)) and runners without symptoms (median at +172% (IQR: 91–393)) (ES: 0.54 (95%CI: 0.07–1.00) (Table 3).

Fluid consumption was higher in runners with lower gastrointestinal symptoms when compared to runners who did not experience these symptoms (Table 4).

### 3.4. Comparison of Biological Parameters According to Race Completion

Runners who forfeited during the race were significantly older than runners who completed the race (medians at 45 years (IQR: 41–50.5) and 42 years (IQR: 39–48), respectively) (Table 4). For runners who forfeited, the run time was shorter than for runners who completed the race (medians at 13 h (IQR: 9–15) and 21 h (IQR: 19–22), respectively) (Table 4).

Pre-race biomarker medians were not significantly different between runners who forfeited and those who completed the race (Table 5).

Runners who did not complete the race had a significantly lower post-race median of AST (ES: −1.23 (95%CI: −1.85–−0.60), LDH activity (ES: −0.94 (95%CI: −1.55–−0.33) and IL-6 (ES: −1.31 (95%CI: −1.93–−0.67) (Table 5).

AST activity variation was different between runners who withdrew (median at +227% (IQR: 115–489)) and runners who completed the race (median at +658% (IQR: 388–1250)) (ES: −1.15 (95%CI: −1.76–−0.52) (Table 5). IL-10 variation was different between runners who withdrew (median at +128% (IQR: 69–155)) and runners who completed the race (median at +633% (IQR: 316–1453)) (ES: −0.99 (95%CI: −1.60–−0.37) (Table 5).

## 4. Discussion

In order to identify a biological marker reflecting exercise-induced lower gastrointestinal symptoms in endurance athletes, we performed an original study of 15 blood biomarkers in a large cohort of ultra-trailers.

About primary endpoint, in our study, the percentage of runners suffering from lower digestive symptoms was 45.5%, in line with data published in the literature concerning the occurrence of these symptoms during running [4]. Unsurprisingly given the intensity of the race, significant variations were observed for all the studied parameters at the end of the event. Cell lysis markers such as LDH, CK, AST and ALT were significantly increased, which is in agreement with data available in the literature [17]. Electrolytes and creatinine variations were also consistent with the context of intracellular dehydration, hypovolemia and secondary hyperaldosteronism [18]. Variations in electrolytes and creatinine were also consistent with a context of intracellular dehydration, hypovolemia and secondary hyperaldosteronism. Indeed, our results may be consistent with possible dehydration due to exertion, as our data collection showed a median water intake of 333 mL/h in our participants, with no details of sodium supplementation, unfortunately. These figures seem relatively low compared with the recommended hydration intake during the race [19]. A significant post-race increase in I-FABP concentration was observed for all runners, and did not discriminate between participants that experienced symptoms and those who did not. Our results are consistent with data in the literature which tend to refute the hypothesis of a correlation between mesenteric ischemia reflected by I-FABP levels and exercise-induced abdominal pain [20,21]. Regarding inflammatory markers, the increased expression of cytokines, including IL-6, IL-10 and IL-1Ra, can be attributed to exercise; indeed, the increase in pro- and anti-inflammatory cytokines has already been demonstrated during physical exercise [9]. This increase in plasmatic concentrations is proportional to exercise intensity, duration [22] and muscle damage involved [23]. Interestingly, among these elevated cytokines, IL-10 allowed the differentiation between participants with lower gastrointestinal symptoms and those without with statistical significance. We did not study markers of bacterial translocation via the disruption of the intestinal barrier, which is also affected by dietary intake and may play a part in the initiation of these symptoms [24]. This bacterial translocation may be at the root of the disturbance in the systemic cytokine profile. The significant increase in IL-10 and IL-1Ra observed in our study may reflect this disruption of the intestinal barrier and translocation. In fact, prolonged running induces transient damage to the intestinal epithelium, increases gastrointestinal symptoms and promotes greater disruption of the systemic cytokine profile [25]. Finally, concerning IL-6, although an increase in this marker in relation to mesenteric ischemia has already been observed [26], it is important to consider the impact of muscle mass in the results’ interpretation [27]. In fact, IL-6 is released from myocytes in response to skeletal muscle contraction, and may reflect muscle mass [28], on the other hand, the lack of data concerning the body composition of athletes during this race did not allow us to assess muscle mass.

A secondary endpoint was to evaluate the evolution of these biomarkers in athletes who did not complete the race. Data from runners who forfeited before the end of the race were analyzed in order to cross-check the results. The increase in IL-6, LDH, AST and CK was significantly less marked in this subgroup, when compared to runners who completed the race. These results demonstrate the observation made previously concerning an increase in these markers which is proportional to the duration and intensity of physical exercise, as well as to the associated muscle damage. Finally, the lower variations in IL-6, LDH, AST and CK observed could suggest lower muscle mass in runners who dropped out of the race, as IL-6 reflects muscle mass during exercise [27,28]. LDH, AST and CK are not only markers of muscular suffering but also activity [16].

The strengths of this study lie in the size of our cohort, in the diversity of the biomarkers studied, as well as the availability of pre-race and post-race samples. This allowed us to highlight differences in kinetics, which is a step in the direction of personalized medicine in sports medicine. The weaknesses of our study are related to the difficulty in collecting certain data such as fluid intake, and the impossibility to monitor athletes for longer periods of time after the race, with potential recourse to imaging examinations in case of doubt in the diagnosis of severe digestive disorders such as mesenteric ischemia. This was not possible, as the runners came from all over France. We were not able to ensure any biological or clinical follow-up. Further investigations must be conducted to elucidate the exact etiology (certainly multi-factorial) of the exercise-induced digestive disorders. Moreover, the data collected on food intake during the race could not be interpreted. It has been shown in the literature that digestive symptoms induced by exercise, and particularly by running, can be linked to food intake, particularly carbohydrate intake. In this study, we were unable to study the carbohydrate intake of individual runners or the pre-race dietary strategy [29]. Lastly, climatic conditions, which may play an important role in this context, were not data collected [25].

## 5. Conclusions

Blood concentrations of I-FABP, D-Lactate, IL-6, IL-10, IL-1Ra, AST, ALT, LDH, CPK, sodium and creatinine are significantly increased after an ultra-trail. We investigated the value of these biomarkers in athletes presenting lower gastrointestinal symptoms after an ultra-trail (primary endpoint) and we evaluated the evolution of these biomarkers in athletes who did not complete the race (secondary endpoint). For the time being, due to certain limitations (athletes not monitored for several days after the race, insufficient data collected on caloric and hydro-sodic intake), there are no practical applications for these markers, but they do support certain physio-pathological hypotheses.

IL-10 and IL-1Ra could be associated with the occurrence of lower gastrointestinal symptoms and may be useful in identifying athletes to be monitored. This observation could be explained by the disruption of the intestinal barrier and consequent bacterial translocation. This bacterial translocation may be at the root of the disturbance in the systemic cytokine profile with the increases in IL-10 and IL-1Ra [25].

Insufficient AST, IL-6 and LDH levels could be one of the causes of withdrawal; in fact, as mentioned in the introduction, the performance in these disciplines is predicted by aerobic capacity, as well as by muscular strength and body composition (not studied there) for distances around 100 km [1]. Basic research studies will help us better understand the role of these biomarkers.

## Figures and Tables

**Table 1:** Description of the population.

Parameters	All Runners Included	Runners with Blood Samples Collected Before and After the Race ^1^	*p*-Value
*n*	120	76	/
Mean age in years (SD)	44 (8)	43 (8)	0.32
Male, *n* (%)	112 (93.3)	72 (94.7)	0.46
Female, *n* (%)	8 (6.7)	4 (5.3)	
Mean weight in kg (SD)	70 (8)	70 (8)	0.58
Mean height in cm (SD)	177 (7)	177 (7)	0.52
Mean body mass index in kg/m^2^ (SD)	22.5 (1.9)	22.4 (1.6)	0.21
Median run time in h (IQR)	19 (15–22)	20 (17–22)	0.22
Mean fluid intake in mL/h (SD)	334 (147)	335 (149)	0.71
Mean cumulative distance over one week in km to estimate training level (SD)	88 (40)	86 (40)	0.23
Lower gastrointestinal symptoms, *n* (%)	55 (45.8)	35 (45.5)	0.61
Forfeited before the end of the race, *n* (%)	36 (30)	13 (16.9)	<0.001

IQR: interquartile range; *n*: number of runners; SD: standard deviation. ^1^ Little’s MCAR test reveals that missing values are MCAR (*p*-value = 0.774).

**Table 2:** Impact of running on blood parameters (*n* = 76).

Blood Parameters (Unit)	Pre-Race Median (Min, Max, IQR)	Post-Race Median (Min, Max, IQR)	*p*-Value
AST activity (UI/L)	17 (8, 85, 13–20)	113 (27, 939, 74–184)	<0.001 ***
ALT activity (UI/L)	20 (9, 56, 16–25)	34 (5, 256, 30–49)	<0.001 ***
CK activity (UI/L)	127 (5, 465, 94–150)	>1300 (441, >1300, >1300–>1300)	<0.001 ***
Creatinine (μmol/L)	80 (59, 109, 74–88)	107 (72, 176, 92–125.6)	<0.001 ***
LDH activity (UI/L)	169 (125, 750, 153–192)	312 (187, 750, 263–389)	<0.001 ***
Sodium (mmol/L)	140 (127, 160, 138–144)	146 (133, 157, 138–146)	0.02 *
Potassium (mmol/L)	4.2 (3.3, 6.2, 4–4.6)	4 (3.32, 5.5, 3.8–4.3)	0.002 **
Chloride (mmol/L)	103 (96, 115.5, 102–105)	105 (98.8, 115, 102–108)	0.02 *
I-FABP (pg/mL)	584 (300, 1712, 458–828)	880 (290, 3169, 628–1308)	<0.001 ***
IL-10 (pg/mL)	0.6 (0.6, 14.8, 0.6–1.5)	5.9 (1.4, 326, 3.31–11.7)	<0.001 ***
IL-1Ra (pg/mL)	263 (144, 665, 222–312)	825 (321, 6000, 582–11,311)	<0.001 ***
IL-6 (pg/mL)	0.87 (0.3, 11.5, 0.7–1.3)	35 (5, 291, 24–55)	<0.001 ***
TNF-alpha (pg/mL)	10 (6, 15, 10–12)	11 (7.3, 20, 10–13)	<0.001 ***
D-Lactate (mmol/L)	5.2 (<0.01, >10, 4–6.8)	7.2 (3.6, >10, 5.6–9)	<0.001 ***

ALT: alanine aminotransferase; AST: aspartate aminotransferase; CK: creatine kinase; I-FABP: intestinal fatty acid-binding protein; IL-1Ra: interleukin-1 receptor antagonist; IL-6: interleukin-6; IL-10: interleukin-10; IQR: interquartile range; LDH: lactate dehydrogenase; max: maximum; min: minimum; TNF-alpha: tumor necrosis factor-alpha. *: *p* < 0.05, **: *p* < 0.01; ***: *p* < 0.001.

**Table 3:** Comparison of biological parameters (pre-race concentration, post-race concentration, delta between pre- and post-race) with the presence or absence of lower gastrointestinal symptoms at the end of the race (*n* = 76).

Blood Parameters (Unit)	Median Pre-Race Concentrations (IQR)		*p*-Values */£	Median Post-Race Concentrations (IQR)		*p*-Values */£	Median Delta in % (IQR)		*p*-Values */£
	With Lower Gastrointestinal Symptoms	Without Lower Gastrointestinal Symptoms		With Lower Gastrointestinal Symptoms	Without Lower Gastrointestinal Symptoms		With Lower Gastrointestinal Symptoms	Without Lower Gastrointestinal Symptoms	
AST activity (UI/L)	17 (12–21)	16.5 (13–20)	0.62/0.21	120 (73–196)	105 (74–184)	0.39/0.26	658 (314–1655)	489 (304–833)	0.12/0.32
ALT activity (UI/L)	20 (16–28)	19.5 (16–25)	0.59/0.55	42 (32–63)	33 (26–38)	0.07/0.18	69 (44–183)	55 (36–106)	0.25/0.29
CK activity (UI/L)	131 (96–166)	118 (89–145)	0.40/0.90	>1300 (>1300–>1300)	>1300 (>1300–>1300)	NA	NA	NA	NA
Creatinine (μmol/L)	81 (75–88)	77 (72–87)	0.26/0.53	106 (93–127)	107 (90–126)	0.96/0.90	41 (12–50)	26 (16–52)	1.00/0.71
LDH activity (UI/L)	171 (154–199)	168 (151–192)	0.78/0.27	331 (282–468)	290 (251–359)	0.06/0.07	105 (57–157)	68 (46–102)	0.05/0.08
Sodium (mmol/L)	141 (140–145)	140 (137–142)	0.02/0.15	142 (138–147)	143 (139–146)	0.44/0.48	0.3 (−2–4.2)	2,6 (0–4.8)	0.13/0.16
Potassium (mmol/L)	4.23 (4–4.6)	4.23 (4–4.6)	0.88/0.98	4 (3.9–4.3)	4 (3.7–4.3)	0.53/0.41	−3.7 (−12–4)	−3.5 (−14–1.7)	0.56/0.62
Chloride (mmol/L)	104 (103–106)	103 (101–105)	0.08/0.26	105 (101–107)	105.5 (103–108)	0.20/0.22	0 (−3.6–5)	2.4 (-0.7–7)	0.09/0.13
I-FABP (pg/mL)	584 (454–821)	569 (460–793)	0.82/0.65	861(617–1253)	925 (640–925)	0.48/0.41	48.4 (7.2–106)	78.4 (0.7–149)	0.45/0.41
IL-10 (pg/mL)	1.31 (0.6–1.6)	0.6 (0.6–1.4)	0.18/0.96	8.7 (4.2–16)	4.8 (2.4–9)	0.01/0.03	591 (291–591)	412 (145–1433)	0.21/0.20
IL-1Ra (pg/mL)	270 (204–319)	256 (225–318)	0.95/0.70	884 (649–1739)	787 (512–1250)	0.10/0.07	231 (169–551)	172 (91–393)	0.04/0.03
IL-6 (pg/mL)	0.9 (0.5–1.3)	0.9 (0.7–1.2)	0.66/0.22	42.3 (25–60.5)	35 (23–52)	0.26/0.19	4560 (2481–8606)	3264 (2090–6468)	0.22/0.16
TNF-alpha (pg/mL)	10.5 (9.3–12)	10.4 (9.8–11.5)	0.67/0.87	11.9 (10–13)	11 (10–13)	0.32/0.18	8.6 (2.5–28)	8.3 (−3–15)	0.29/0.15
D-Lactate (mmol/L)	5.4 (4–9)	5 (4.2–6.1)	0.17/0.07	7.4 (6–9)	7.1 (4.7–8.8)	0.43/0.45	35 (−15–86)	37.5 (2.3–104)	0.48/0.55

ALT: alanine aminotransferase; AST: aspartate aminotransferase; CK: creatine kinase; I-FABP: intestinal fatty acid-binding protein; IL-1Ra: interleukin-1 receptor antagonist; IL: interleukin; IQR: interquartile range; LDH: lactate dehydrogenase; NA: not applicable; TNF-alpha: tumor necrosis factor-alpha. *: *p*-value after univariate analysis; £: *p*-value after multivariate analyses conducted with multiple logistic regression to take into account sex and age.

**Table 4:** Clinical characteristics according to presence of lower gastrointestinal symptoms or retirement before the end of the race.

	Lower Gastrointestinal Symptoms		*p*-Value	Race Completion		*p*-Value
	Present	Absent		No	Yes	
*n*	35	41		36	84	/
Median age in years (IQR)	43 (40–49)	40.5 (37.5–47)	0.12	45 (41–50.5)	42 (39–48)	0.04
Male, *n* (%)	34 (97)	38 (93)	0.62	33 (92)	79 (94)	0.23
Median body mass index in kg/m^2^ (IQR)	23 (21–24)	22 (21–24)	0.38	23 (22–24)	22 (21–24)	0.28
Median fluid intake in mL/h (IQR)	334 (271–457)	281 (224–354)	0.05	270 (115–629)	320 (259–409)	0.62
Median run time in hour (IQR)	20 (18–21)	20 (16–23)	0.93	13 (9–15)	21 (19–22)	<0.001
Median cumulative distance over one week in km to estimate training level (IQR)	77 (53–100)	93 (74–100)	0.31	83 (70–112)	80 (60–100)	0.22

IQR: interquartile range; *n*: number of runners.

**Table 5:** Comparison of biological parameters (pre-race concentration, post-race concentration, delta between pre- and post-race) according to race completion (*n* = 76).

Blood Parameters (Unit)	Median Pre-Race Concentrations (IQR)		*p*-Values */£	Median Post-Race Concentrations (IQR)		*p*-Values */£	Median Delta in % (IQR)		*p*-Values */£
	Race Forfeited	Race Completed		Race Forfeited	Race Completed		Race Forfeited	Race Completed	
AST activity (UI/L)	17 (12.5–20)	16 (13–20.5)	0.98/0.85	54 (49–96)	121 (88–205)	<0.001 */0.004 *	227 (115–489)	658 (388–1250)	<0.001 */0.008 *
ALT activity (UI/L)	20.5 (18–26)	20 (16–25)	0.29/0.82	33 (27–38)	35 (30–55)	0.22/0.12	29 (19–65)	72 (44–140)	0.08/0.03 *
CK activity (UI/L)	99.5 (83–143)	122 (96–150.5)	0.09/0.62	>1300 (1110–>1300)	>1300 (>1300–>1300)	NA	NA	NA	NA
Creatinine (μmol/L)	80.6 (75–59)	80 (73–88)	0.86/0.90	88 (79–93)	111 (97–127)	0.001 */0.15	16 (−1–24)	40 (19–52)	0.003 */0.34
LDH activity (UI/L)	178.5 (163.5–202)	165.5 (150.5–188)	0.06/0.97	253 (242–288)	326 (280–407)	0.002 */0.02 *	43 (35–57)	93 (59–142)	0.002 */0.06
Sodium (mmol/L)	141 (139–144)	140 (139–143)	0.49/0.46	141 (139–145)	143 (139–146)	0.40/0.28	1 (0.1–5)	2 (−2–5)	0.93/0.91
Potassium (mmol/L)	4.22 (4–4.4)	4.23 (4–4.6)	0.81/0.86	4.1 (3.6–4.3)	4 (3.8–4.3)	0.90/0.50	−1 (−14–25)	−4 (−13–3)	0.81/0.47
Chloride (mmol/L)	103 (102–106)	103 (102–105)	0.46/0.38	104 (102–106)	105 (102.5–108)	0.35/0.18	1.7 (0–5)	1 (−1.8–5)	0.98/0.73
I-FABP (pg/mL)	583 (432–752)	561 (420–797)	0.90/0.95	679 (526–968)	905 (651–1531)	0.03 */0.09	14.3 (−25–94)	74.2 (7.2–140)	0.04 */0.10
IL-10 (pg/mL)	1.3 (0.6–1.5)	1.2 (0.6–1.5)	0.85/0.73	2.3 (2–3.3)	7.8 (4.3–12)	<0.001 */0.13	128 (69–155)	633 (316–1453)	<0.001 */0.03 *
IL-1Ra (pg/mL)	300 (245–340)	258 (211–319)	0.06/0.98	747 (581–894)	876 (606–1398)	0.34/0.50	148 (98–229)	226 (128–423)	0.14/0.75
IL-6 (pg/mL)	0.8 (0.7–1.2)	1 (0.7–1.3)	0.51/0.55	13.6 (9.7–33.7)	39 (29–58)	0.002 */0.03 *	1529 (987–4266)	4455 (2573–8151)	0.006 */0.82
TNF-alpha (pg/mL)	10.3 (9.4–11)	10.2 (9.3–11)	0.86/0.67	12.4 (10–13)	11 (10–13)	0.29/0.06	13 (1.6–21)	8 (0–24)	0.43/0.41
D-Lactate (mmol/L)	5.6 (4–7)	5.3 (4–7)	0.36/0.31	7.2 (6.5–8.4)	7.2 (5.4–8.8)	0.93/0.43	0 (−10–23)	49 (0.6–104)	0.04 */0.13

ALT: alanine aminotransferase; AST: aspartate aminotransferase; CK: creatine kinase; I-FABP: intestinal fatty acid-binding protein; IL-1Ra: interleukin-1 receptor antagonist; IL-6: interleukin-6; IL-10: interleukin-10; IQR: interquartile range; LDH: lactate dehydrogenase; max: maximum; min: minimum; NA: not applicable; TNF-alpha: tumor necrosis factor-alpha. *: *p*-value after univariate analysis; £: *p*-value after multivariate analyses conducted with multiple logistic regression to take into account sex, age and run time (run time for comparisons concerning post-race concentrations and delta %).

## Data Availability

Individual participant data (including data dictionaries) will be made available. However, the French National Commission on Data Protection (CNIL) prohibits the release of data without prior consent. Therefore, the data underlying the study results cannot be made freely available due to ethical and legal restrictions. However, de-identified individual participant data underlying the results reported in the manuscript (text, tables) can be requested from the COMBINE Steering Committee. Interested researchers should contact dbouvier@chu-clermontferrand.fr to request access to the data. In addition to individual participant data, the statistical analysis plan will also be available. Data will be available 6 months after publication of the article, with no end date specified. The data will be available to any researcher who requests them, for any type of analysis. All requests will be evaluated for scientific relevance. Data access will be granted only after scientific evaluation and the signing of a data-sharing agreement. This agreement must specify the type of data requested and must be signed between the applicants and the sponsor, the Clermont-Ferrand University Hospital.
